# No intrinsic gender differences in children’s earliest numerical abilities

**DOI:** 10.1038/s41539-018-0028-7

**Published:** 2018-07-06

**Authors:** Alyssa J. Kersey, Emily J. Braham, Kelsey D. Csumitta, Melissa E. Libertus, Jessica F. Cantlon

**Affiliations:** 1 Department of Brain and Cognitive Sciences, University of Rochester, Rochester, NY 14627 USA; 2 Rochester Center for Brain Imaging, University of Rochester Medical Center, Rochester, NY 14620 USA; 3 Department of Psychology, University of Pittsburgh, Pittsburgh, PA 15260 USA; 4 Learning Research and Development Center, University of Pittsburgh, Pittsburgh, PA 15260 USA

## Abstract

Recent public discussions have suggested that the under-representation of women in science and mathematics careers can be traced back to intrinsic differences in aptitude. However, true gender differences are difficult to assess because sociocultural influences enter at an early point in childhood. If these claims of intrinsic differences are true, then gender differences in quantitative and mathematical abilities should emerge early in human development. We examined cross-sectional gender differences in mathematical cognition from over 500 children aged 6 months to 8 years by compiling data from five published studies with unpublished data from longitudinal records. We targeted three key milestones of numerical development: numerosity perception, culturally trained counting, and formal and informal elementary mathematics concepts. In addition to testing for statistical differences between boys’ and girls’ mean performance and variability, we also tested for statistical equivalence between boys’ and girls’ performance. Across all stages of numerical development, analyses consistently revealed that boys and girls do not differ in early quantitative and mathematical ability. These findings indicate that boys and girls are equally equipped to reason about mathematics during early childhood.

## Education: No gender differences in children’s mathematics

Girls and boys show no cognitive differences in mathematical ability during infancy and early childhood across multiple tasks. To compare boys’ and girls’ early mathematical thinking, Alyssa Kersey and colleagues at the University of Rochester and University of Pittsburgh examined performance on three milestones of numerical development: numerosity sensitivity, counting range, and early mathematics achievement. Researchers tested not only for statistical differences between boys and girls but also statistical similarities. Across all aspects of early mathematics development, boys and girls exhibited no statistical differences and instead generally showed statistical equivalence. The results suggest that girls and boys begin development with an equivalent cognitive capacity for mathematics.

## Introduction

Adult gender differences in science, technology, engineering, and math (STEM) career representation sometimes are thought to originate from inborn differences between the sexes in aptitude for STEM fields.^1–5^ Gender differences could be biological differences that are present at birth, or they might emerge over time with maturation.^4^ In this study, we focus on gender differences in early childhood. Although adult STEM talent is derived from a large suite of cognitive abilities and unlikely to be traceable to a single domain or skill, if intrinsic differences between the sexes are indeed a root cause for the under-representation of women in STEM, one expectation is that gender differences in quantitative cognition will emerge early in human development.

Understanding the nature of gender differences in mathematics has been a focus of research for many years. However, differences in measurements, analyses, and participant samples have led to a variety of findings. For one, differences can emerge in mean performance on mathematical tasks,^6–8^ and small differences in favor of boys have been reported in a range of numerical skills by the end of kindergarten.^9^ Although most studies of school-aged children that find gender differences report higher performance in boys, some studies have only found advantages for boys when tasks involve more reasoning or are more spatial in nature.^2,10^ In contrast, elementary school girls sometimes show an advantage on computational tasks and when performance is assessed using school grades.^11^ Other studies find no differences, trivial differences, or differences in older children, but not younger children.^10,12–14^ Group differences can sometimes be attributed to cohort effects. For instance, some studies show that differences between US and Chinese children in mathematics depend on generation or school,^15,16^ and a recent study showed that the strength of any advantage in mathematics for boys vs. girls varies by country.^17^ Gender differences may also emerge in the variability of mathematical performance across boys and girls. When these gender differences in cognition are observed, boys tend to show greater variability than girls, resulting in more boys than girls at the high-performing and low-performing ends of distributions.^6–8,17,18^ This may cause gender differences in mean performance to be absent at the group level^12,14^ but detectable at the high-performing and low-performing ends of the distributions.^18^

Another major obstacle in assessing such gender differences on school-based mathematics metrics is that sociocultural influences, such as stereotype threat and the influence of teachers and parents, make it difficult to tease apart gender differences in experience from differences in intrinsic abilities.^19–29^ For example, school-aged children could show gender differences in mathematics abilities because girls are given less or different exposure to mathematics than boys or are told that “math is not for girls.” Therefore, it is unclear whether differences in mathematics abilities are rooted in intrinsic differences in numerical reasoning in early childhood or whether gender differences emerge as a result of differences in cultural exposure to mathematical concepts. Understanding the sources of any gender differences is crucial for optimizing early childhood math and science curricula.

**Fig. 1:**
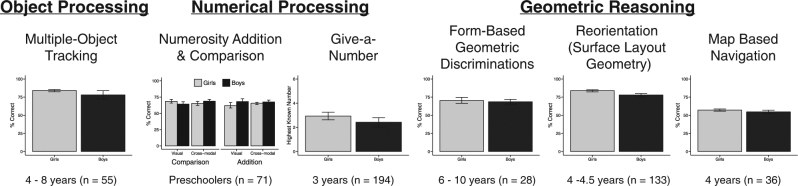
Previously described gender similarities. Redrawn data^30^ showing no gender differences in early childhood on measures of object processing, numerical processing, and geometric reasoning

Previous research^30^ described evidence against the existence of gender differences in visuospatial reasoning in early childhood. Across six tasks, boys and girls performed similarly on measures of object tracking (the ability to follow multiple, independent moving objects), early numerical processing, and core geometric abilities (Fig. 1). Those data revealed no gender differences in some basic cognitive abilities of children aged 3–10 years. However, that research leaves open key areas for investigating gender differences in core numerical processing, including patterns of looking at quantitative information during infancy, early discrimination acuity during quantity processing, and formal mathematics learning.

We examined children’s early mathematical cognition during infancy and early childhood to provide insight into whether gender differences are evident in early childhood. With the exception of the infant data, these data were collected as part of standard testing batteries measuring numerical processing skills. While we acknowledge that there are other ways to measure mathematical thinking in this age range, we combined published data^31–35^ with unpublished data from our longitudinal records that measured children’s performance in three key areas of numerical processing from our standard testing battery of early childhood numerical cognition. First, we assessed numerosity perception and acuity in infancy and childhood. Numerosity perception allows us to estimate the quantity of a set without knowing exactly how many items are in the set—we measured children’s acuity to detect differences in numerosity. Next, we examined two aspects of verbal counting acquisition during preschool, which is the earliest emerging exact understanding of quantities. Finally, we evaluated school-based mathematics during the first few years of schooling when children learn to manipulate numbers. School-based mathematics refers to comprehensive, standardized testing of a variety of mathematical skills including counting proficiency, numeral knowledge, concrete set comparison and transformation, word problems with numerical comparisons and basic arithmetic transformations, and part-whole concepts. Because the tests are age-based, the tasks completed by each child varied. These data are largely unpublished but were combined with published data^31–35^ in order to examine gender differences in over 500 children.

We conducted several analyses to test for statistical differences and statistical equivalence in performance, the emergence or disappearance of differences with age, and statistical differences in variability between groups. Similarities and differences between boys’ and girls’ performance were assessed using independent-samples *t* tests to identify *statistical differences* in mean performance and Schuirmann’s two one-sided tests of equivalence^36^ to identify *statistical equivalence* in mean performance (similarity within ½ standard deviation (s.d.) of the group data; implementation of this test for SAT-Math scores.^37^) Testing for both statistical differences and statistical equivalence is important. Non-significant *t* tests only allow us to conclude that there is not enough evidence to reject the assumption that performance is equivalent between groups. However, this does not necessarily mean that the groups are statistically equivalent. By including tests of equivalence, we can determine whether the lack of a significant difference between groups reflects statistically equivalent distributions of scores between groups. To date, tests of equivalence have not been conducted on data on mathematical abilities in early childhood, but these tests are especially important for informing the “Gender Similarities Hypothesis.”^38,39^ To determine whether the results of the *t* test were consistent across age, we also conducted simultaneous linear regressions with age, gender, and their interaction entered as predictors. A main effect of gender would suggest that there is a difference between boys and girls when controlling for age and an interaction would suggest that differences may emerge only at one end of the age range. In addition to assessing children’s mean performance, we determined whether boys and girls showed equal *variance* in performance using Levene’s test. Testing for equality of variance is particularly important in light of previous work that suggests that there are more high-performing and low-performing males than females because males show greater variability in measures of quantitative processing.^4^ For thoroughness, tests of statistical equivalence and differences in variability on scores controlled for age are reported in Supplement 1 (statistical differences in age-controlled scores should be evident in the regression analyses). Finally, for visualization purposes, we calculated growth curves at the group level following previous work.^40^ Because these curves were calculated at the group level, we do not statistically test for differences between boys’ and girls’ growth rates and simply provide these curves as a way to visualize changes in performance with age.

Across all three aspects of early mathematical cognition assessed here, we would expect that if boys and girls truly differ in their capacities for numerical processing, we should find evidence of statistical differences in mean performance (independent-samples *t* tests), and we should see that this effect is consistent across age (main effect of gender in the linear regressions) or driven by one end of the age range (interaction between gender and age in the linear regression). However, the cross-sectional analysis indicates that there are no robust gender differences in early numerical processing including preverbal numerosity perception, counting acquisition, and school-based mathematics ability.

### Core numerosity perception

Humans have the ability to perceptually estimate the numerical magnitude of a set of objects without counting. For example, without counting, people can rapidly determine that a set of 20 objects is numerically greater than a set of 10. Because numerosity representations are only noisy estimations of number, discrimination between quantities depends on the numerical ratio of the sets based on Weber’s law.^41^ For example, using estimation it is equally easy for people to choose the larger quantity of 10 vs. 5 as 20 vs. 10, because they have the same ratio (2:1 ratio)—quantities with finer ratios like 7 vs. 5 or 15 vs. 10 will be more difficult to discriminate. Research has shown that this ability to represent and discriminate numerosities emerges within the first year of life^42–45^ and that it is evident in nonhuman animals,^46–51^ suggesting an evolutionarily primitive origin. At 6 months, human infants can discriminate quantities that differ by a ratio of 2:1 (e.g., 16 vs. 8 dots),^44,45^ but by 9 months, infants can discriminate quantities at a 3:2 ratio.^45^ Numerosity representations become more refined with age such that 4-year-old children can discriminate at a 4:3 ratio and adults can discriminate at a ratio of 10:9.^52^ The visuospatial nature of numerosity perception makes it an important ability to investigate in children because gender differences in mathematics have sometimes been attributed to fundamental visuospatial skills, such as mental rotation.^53^ Moreover, because the acuity of these representations has been shown to relate to math ability^54–56^ (but note opposing views^57^), understanding whether there are gender differences in early numerical processing is essential to understanding the fundamental nature of gender differences in math achievement. Here we examined data from infants, preschool children, and early school-aged children.

**Fig. 2:**
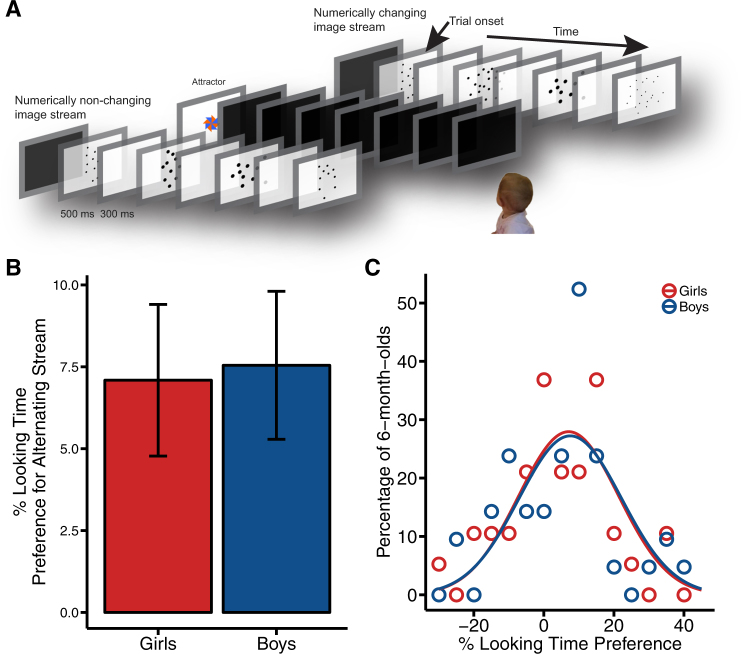
Infant numerosity. **a** Depiction of numerosity change detection task. **b** Average percentage of time looking at the numerically changing image stream for girls (red) and boys (blue). Error bars represent standard error of the mean. **c** Density distributions for percentage of girls (red) and boys (blue) at a given % looking time preference

To test for gender differences in numerosity representations in infancy, we analyzed previously published data from 80 6-month-old infants^35^ (range = 5 months 13 days–6 months 17 days, 38 girls, 42 boys). The precision of infants’ numerosity representations was assessed using a preferential looking paradigm in which infants were presented with two image streams: one in which numerosities alternated between images and one in which numerosity was constant (see Fig. 2a). Infants preferred to look to the numerically alternating image stream if numerosities differed by at least a 2:1 ratio, and there were individual differences in infants’ preferences.^35^ An independent-samples *t* test and Schuirmann’s test of equivalence revealed no gender differences (*t* test: *t*(78) = 0.14, *p* = 0.89, difference = 0.41%, 95% confidence interval (CI) = −6 to 7; equivalence test: *t*_1_(78) = 2.36, *p* = 0.01; *t*_2_(78) = −2.08, *p* = 0.026), with boys showing a mean preference for the numerically alternating image stream of 7.5% (±14.6) and girls showing a mean preference of 7.09% (±14.3). Levene’s test of Equality of Variances revealed no significant differences in variance between girls and boys (*F*(1, 78) < 0.01, *p* = .94, boys’ s.d. = 14.65, girls’ s.d. = 14.27). This is consistent with the previous work showing no overall differences between boys’ and girls’ sensitivity to numerosity in infancy.^58–60^

We also tested for gender differences in numerosity perception in the earliest years of formal education. Two hundred forty-one scores were collected from 3- to 7-year-old children (mean age = 5.48 years, 125 girls, 116 boys; data from 68 children have been previously reported^31,32^). All children completed a computerized numerical comparison task. In this task, children were shown two side-by-side dot arrays and were asked to choose the side that had more dots. The numerical ratio between dot arrays varied between 4:1 and 10:9. This type of numerical discrimination task permits a psychophysical evaluation of numerosity representation and is consistent with previous literature using this task in adults and children.^52,61–65^ Furthermore, performance on this type of task has been shown to be similar to neural measures of numerosity encoding,^31,63^ indicating that this is a fundamental aspect of numerical cognition. Although previous work found that women and girls performed better than men and boys,^52^ sample sizes were small (*n* = 16 per age group), so it is unclear whether these differences are representative of the general population.

**Fig. 3:**
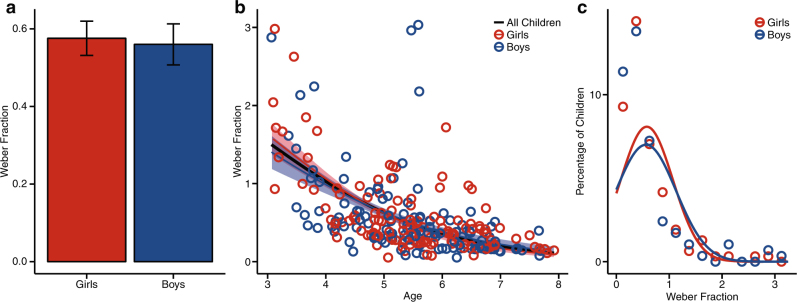
Early childhood numerosity. **a** Average Weber fraction for girls (red) and boys (blue). Error bars represent standard error of the mean. **b** Growth curves for Weber fractions calculated across girls (red), boys (blue), and all children (black). Lightly shaded areas around girls’ and boys’ growth curves indicate 1 standard deviation above and below the mean growth curve. **c** Density distributions for percentage of boys (blue) and girls (red) at a given Weber fraction

To assess the acuity of boys’ and girls’ numerosity representations, Weber fractions (*w*) were calculated for each child.^66^ The *w* score represents the acuity of numerosity representations such that a smaller *w* indicates greater acuity. An independent-samples *t* test and Schuirmann’s equivalence test revealed that boys and girls showed equal acuity of numerosity representations in early childhood (Fig. 3; *t* test: *t*(239) = 0.23, *p* = 0.82, boys’ mean = 0.56, girls’ mean = 0.58, difference = 0.02, 95% CI = −0.12 to 0.15; equivalence test: *t*_1_(239) = 4.10, *p* = 0.00003; *t*_2_(239) = −3.64, *p* = 0.0002). A simultaneous regression further revealed that while acuity improves with age, the effect of age on acuity of numerosity representations does not differ between boys and girls (*F*(3, 237) = 32.03, *p* < 0.0001, *R*^2^ = 0.29; Gender: b = 0.10, *t*(237) = 0.33, *p* = 0.74; Age: *b* = −0.27, *t*(237) = −7.33, *p* < 0.0001; Age × Gender: *b* = 0.02, *t*(237) = 0.30, *p* = 0.77). Finally, Levene’s test of Equality of Variances did not reveal a difference in variance between boys and girls (*F*(1, 239) = 0.09, *p* = 0.76; boys’ s.d. = 0.57, girls’ s.d. = 0.49).

Taken together, we find that from infancy into early childhood, boys and girls do not differ in their earliest numerosity perceptions. Boys and girls are equally capable of discriminating numerosities.

### Culturally trained counting

Verbal counting is the first culturally trained symbolic mathematics concept to develop in children. Knowledge of the verbal counting routine emerges gradually between the ages of 2 and 5 years. First, children learn to rote recite the count list (2–2.5 years). Over the next 6 to 12 months, children begin to acquire the meanings of the number words one at a time: they learn that the number word “one” corresponds to exactly one item, then that the word “two” corresponds to exactly two items, then “three,” and finally “four.” Around 3.5 years, children seemingly suddenly become cardinal-principle knowers in that they learn that each number word refers to a specific quantity and that a number word can be used to label the size of a set as determined by counting.^67–71^ We tested children’s knowledge of the rote-memorized counting sequence with the “How High?” task, and we tested their cardinal knowledge of number and counting principle knowledge with the “Give-N” task.^69,70^ Although there are other ways to assess counting skills and knowledge of the cardinal principles,^9,69–72^ these tasks are commonly used and standardized across the literature. These two measures of culturally trained counting allowed us to determine whether boys or girls show a general advantage for early number word learning or whether there are different patterns of gender differences in memorizing the counting sequence (“How High” task) vs. learning the meanings of number words (“Give-N” task). A general advantage for early number word learning would be supported by differences in favor of one gender on both measures of early number word knowledge. An advantage on only one test would suggest the advantage is isolated to a specific skill.

**Fig. 4:**
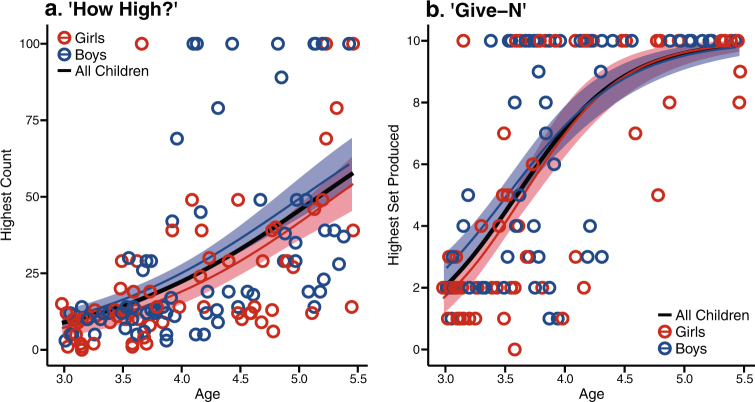
Early childhood counting. Growth curves for performance on the **a** “How High?” task and **b** “Give-N” task. Growth curves are calculated across girls (red), boys (blue), and all children (black). Lightly shaded areas around boys’ and girls’ growth curves indicate 1 standard deviation above and below the mean growth curve

For the “How High?” task, children were asked to count as high as they could until they reached 100. One hundred forty-three children aged 2–5.5 years old were tested (mean age = 4.10 years, 71 girls, 72 boys). An independent-samples *t* test revealed that boys and girls did not show a difference in their ability to memorize the verbal counting sequence (*t*(141) = 1.48, *p* = 0.14, boys’ mean = 30 girls mean = 23, difference = 7, 95% CI = −2 to 16), and Schuirmann’s tests of equivalence found marginal statistical equivalence (*t*_1_(141) = 4.48, *p* = 0.00001; *t*_2_(141) = −1.52, *p* = 0.06). A simultaneous regression confirmed that although children’s ability to recite the count list improves across age, differences do not emerge when controlling for age or at one end of the age range (Fig. 4a for scatterplot of data by age; *F*(3, 139) = 20.96, *p* < 0.0001, *R*^2^ = 0.31; Gender: *b* = 12.75, *t*(139) = 0.58, *p* = 0.56; Age: *b* = 18.26, *t*(139) = 5.10, *p* < 0.0001; Age × Gender: *b* = 4.20, *t*(139) = 0.80, *p* = 0.43). Furthermore, Levene’s test of Equality of Variances revealed no difference in variability (*F*(1, 141) = 1.41, *p* = 0.24; boys’ s.d. = 30, girls’ s.d. = 25). Taken together, this suggests that from 2 to 5.5 years of age, boys and girls show equal proficiency in memorizing and reciting the count list.

Performance on the “How High?” task only represents verbal learning of the culturally trained, rote-memorized list of count terms and is not an index of children’s quantitative or logical reasoning during counting. To test children’s understanding of the counting procedure, we tested children on the “Give-N” task. In the “Give-N” task,^69,70^ children were asked to count in order to produce sets of 1 to 10 objects. One hundred and twenty-three children aged 2.98–5.47 years completed the tasks (mean age = 3.87 years, 65 girls, 58 boys). Children were scored by the highest set size that they could correctly produce. An independent-samples *t* test revealed no statistical difference between boys and girls, but Schuirmann’s tests of equivalence test failed to find statistical equivalence (*t* test: *t*(121) = 1.67, *p* = 0.097, boys’ mean = 6.38, girls’ mean = 5.26, difference = 1.12, 95% CI = −0.2 to 2.44; equivalence tests: *t*_1_(121) = 4.46, *p* = 0.00001; *t*_2_(121) = −1.12, *p* = 0.13). The simultaneous regression revealed a main effect of age, but no effect of gender or interaction between gender and age (Fig. 4b for scatterplot of data by age. *F*(3, 119) = 31.63, *p* < 0.0001, *R*^2^ = 0.44; Gender: *b* = 1.67, *t*(119) = 0.57, *p* = 0.57; Age: *b* = 3.58, *t*(119) = 7.45, *p* < 0.0001; Age × Gender: *b* = 0.23, *t*(119) = 0.30, *p* = 0.76). In addition, we did not detect differences in variance between boys and girls (*F*(1, 121) = 0, *p* = 0.99; boys’ s.d. = 3.61, girls’ s.d. = 3.78). Overall, there are no strong differences between boys and girls in their ability to use counting to produce sets.

Thus, boys and girls do not significantly differ in their cardinal and logical knowledge of the counting sequence during early childhood. The lack of a difference between boys and girls is consistent with the findings depicted in Fig. 1 that tested 194 3-year-old children on similar counting tasks.^30^

In sum, we find that boys and girls show equal proficiency in memorizing and reciting the count list, and comparable abilities to learn the logic of the counting sequence. We conclude that there is no true gender difference in children’s early counting.

### Formal and informal early elementary mathematics

Children begin to learn school-based numerical and mathematical concepts shortly after acquiring the counting principles. To test for early gender differences in the foundations of school-based mathematical concepts, we administered the Test of Early Mathematics Ability Third Edition (TEMA-3^73^) to 275 children aged 3.07–7.92 years (mean age = 5.45 years, 133 boys, 142 girls; data from 77 children have been previously reported^32–34^). The TEMA-3 is a comprehensive test of school-based mathematical knowledge for children aged 3–9 years. Items are categorized as “formal” and “informal”: Formal items tap into knowledge that is formally taught such as numeral names, numeral writing, and arithmetic facts. Informal items tap into children’s abilities to count and reason about quantitative relations and transformations that draw on acquired knowledge but are not explicitly trained or memorized. Although some test items overlap with the skills measured in the previous section on verbal counting acquisition, the TEMA-3 represents math achievement at a broader level. Importantly, the achievement scores that result from the TEMA-3 reflect knowledge on a wide range of mathematical skills including, but not limited to, counting ability. We compared boys’ and girls’ performance on the TEMA-3 overall and on items tapping into formal vs. informal math achievement separately.

**Fig. 5:**
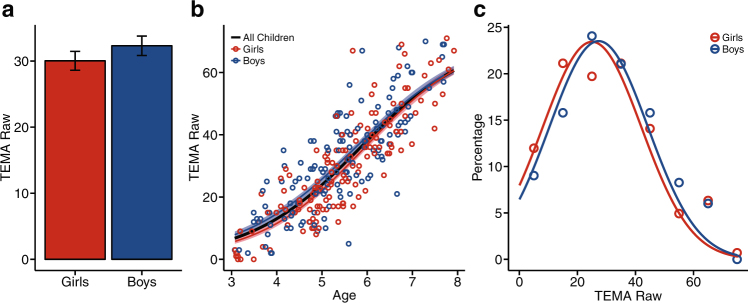
Early childhood mathematics. **a** Average raw TEMA score for girls (red) and boys (blue). Error bars represent standard error of the mean. **b** Growth curves for performance on TEMA calculated across girls (red), boys (blue), and all children (black). Lightly shaded areas around boys’ and girls’ growth curves indicate 1 standard deviation above and below the mean growth curve. **c** Density distributions for percentage of girls (red) and boys (blue) at a given raw TEMA score

Boys and girls did not differ in overall math achievement, suggesting that children show equal understanding of math concepts in early childhood (Fig. 5; *t* test: *t*(273) = 1.11, *p* = 0.27, boys’ mean = 32.32, girls’ mean = 30.04, difference = 2.28, 95% CI = −1.76 to 6.31; equivalence test: *t*_1_(273) = 5.25, *p* < 0.001; *t*_2_(212) = −3.04, *p* = 0.001; test of equality of variances: *F*(1, 273) = 0.002, *p* = 0.99; boys’ s.d. = 16.96 girls’ s.d. 17.02). This pattern was consistent across age suggesting that during early childhood boys and girls show equal competency for math concepts (regression: *F*(3, 271) = 224.3, *p* < 0.00001, *R*^2^ = 0.71; Gender: *b* = 3.81, *t*(271) = 0.70, *p* = 0.49; Age: *b* = 12.72, *t*(271) = 19.00, *p* < 0.0001; Gender × Age: *b* = 0.19, *t*(271) = 0.19, *p* = 0.85).

**Fig. 6:**
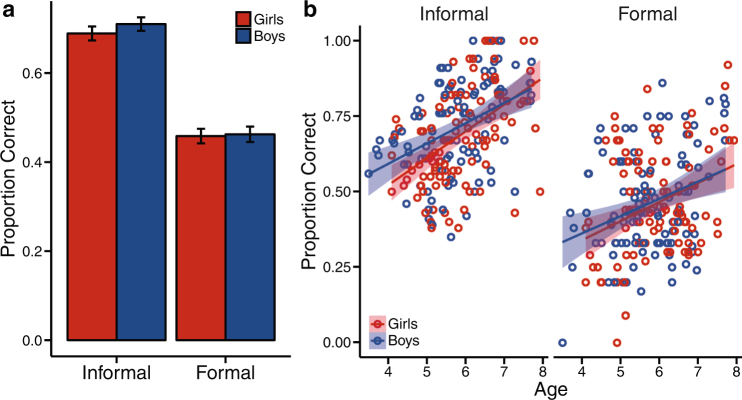
Early childhood formal and informal mathematics. **a** Average proportion correct (mean score) for administered TEMA informal and formal questions for girls (red) and boys (blue). Error bars represent standard error of the mean. **b** Proportion correct (mean score) for administered Informal and Formal Questions plotted by age. Lightly shaded areas around the regression lines indicate the 95% confidence interval

To look at differences in boys’ and girls’ performance by question type, we compared formal vs. informal math scores. We conducted a 2 (Formal/Informal) × 2 (Boys/Girls) repeated-measures analysis of variance (ANOVA) on a subset of the data from children who answered at least four formal questions and at least four informal questions (for a similar approach^74^). We found no interaction between gender and question type nor did we find a main effect of gender (Fig. 6; Gender: *F*(1, 207) = 0.56, *p* = 0.46; Question Type: *F*(1, 207) = 235.98, *p* < 0.0001; Gender × Question Type: *F*(1, 207) = 0.30, *p* = 0.58). Furthermore, we found statistical equivalence between boys’ scores and girls’ scores and no differences in variances for both formal and informal questions (Formal Questions: equivalence tests: *t*_1_(207) = 3.77, *p* = 0.0001; *t*_2_(107) = −3.44, *p* = 0.0003, boys’ mean = 0.46, girls’ mean = 0.46; variance test: *F*(1, 207) = 0.24, *p* = 0.63, boys’ s.d. = 0.17, girls’ s.d. = 0.17; Informal Questions: equivalence tests: *t*_1_(207) = 4.57, *p* < 0.00001; *t*_2_(207) = −2.65, *p* = 0.004, boys’ mean = 0.71, girls’ mean = 0.69; variance test: *F*(1, 207) = 0.32, *p* = 0.57; boys’ s.d. = 0.15, girls’ s.d. = 0.16). In addition, differences did not emerge for either question type when controlling for age or testing for interactions between gender and age (Formal Questions: *F*(3, 205) = 8.25, *p* = 0.00003, *R*^2^ = 0.12; Gender: *b* = 0.05, *t*(205) = 0.14, *p* = 0.74; Age: *b* = 0.06, *t*(205) = 3.70, *p* = 0.0003; Gender × Age: *b* = −0.01, *t*(205) = −0.25, p = 0.81; Informal Questions: *F*(3, 205) = 19.62, *p* < 0.0001, *R*^2^ = 0.22; Gender: *b* = 0.16, *t*(205) = 1.29, *p* = 0.20; Age: *b* = 0.09, *t*(205) = 6.09, *p* < 0.00001; Gender × Age: *b* = −0.02, *t*(205) = −1.05, *p* = 0.29).

In sum, we did not find any robust performance differences in early childhood math ability between boys and girls. Differences did not emerge with age or by question type. This suggests that boys and girls show equal competency forming mathematics concepts in early childhood.

## Discussion

Recent public discussions surrounding the under-representation of women in STEM fields have suggested that differences in career choices between men and women could be due to intrinsic differences in aptitude in STEM domains. This claim would predict that gender differences should be evident from early on in childhood. Our data, compiled across studies from over 500 infants and children, provide a comprehensive analysis of the effect of gender on early mathematical cognition, and show that in fact, there are no substantive gender differences in mathematical thinking skills during infancy or early childhood. Boys and girls perform equivalently on numerosity perception, counting acquisition, and early school-based math concepts. Our results are consistent with those of a previous study of nearly 200 children who were tested on knowledge of the counting procedure using the “Give-N” task and found no evidence of a statistical difference between boys and girls.^30^ Furthermore, early school-based mathematical concepts that build upon knowledge of the logical principles of counting did not show any gender-based differences, suggesting that boys and girls learn mathematics similarly even beyond counting acquisition, into early schooling. This interpretation is consistent with a prior analysis of three million elementary school children showing that school test performance differences in mathematics between boys and girls are non-existent or trivial during elementary school, but steadily increase through high school and college.^10,13^ Thus, boys and girls begin education with equivalent early mathematical thinking skills.

Although these results are consistent with some previous work in this age range,^10,12,30^ these results contrast with other work in this age range. For example, a small advantage for boys in a variety of numerical skills by the end of kindergarten has been previously reported.^9^ However, the growth curve trajectories they fit for each test suggest that these differences were not consistent across every timepoint assessed during kindergarten. For some tests, such as numerical estimation and counting skills, boys and girls were indistinguishable at the initial timepoint. For other tests, such as patterns, number recognition, and number combinations, boys and girls had overlapping scores in the middle timepoints. This shows that even when gender differences are detected, they are inconsistent and highlights the importance of future work that measures gender differences using a longitudinal approach. In contrast, their work found consistent differences in math ability based on socioeconomic status.^9^ Although gender differences between socioeconomic statuses could not be assessed in the present study, it is important to take this into consideration in future work. Comparing the present study to previous work also emphasizes the reality that there are many ways to measure mathematical thinking in early childhood and group differences could vary across tasks, cohorts, and age.

The absence of statistical differences across the major developmental milestones of early mathematical cognition are unlikely to be due to sample size. Power analyses suggest that given the sizes of the samples analyzed here, we should have been able to detect small to medium effect sizes ranging from Cohen’s *d* = 0.34 to 0.65 (80% power, *p* = 0.05; Infant Numerosity Comparison (looking time): *d* = 0.65; Early Childhood Numerosity Comparison (*w*): *d* = 0.37; Recitation of Count List (“How High?” task): *d* = 0.47; Counting Principles (“Give-N” task): *d* = 0.52; Math Concepts (TEMA): *d* = 0.34, (Formal/Informal Math Scores): *d* = 0.40). Importantly, even if smaller effects do exist, they are unlikely to reliably, meaningfully, or consistently manifest in children. Caution should be taken when interpreting any small effects in large sample to ensure that their importance is not over-exaggerated.^13,75^

The origin of adult gender differences in science, technology, engineering, and mathematics likely has a complex sociological explanation^2,4^ and cannot be easily reduced to intrinsic differences in aptitude in early childhood. Women have been discouraged from participating in mathematics and science, and there is a long legacy of sexism in academics. Stereotype threat has been shown to have deleterious effects on girls’ and women’s mathematics performance^19,20^ (but see Ganley et al.^76^), and the strength of implicit stereotypes associating men over women with science predicted gender differences in 8th grade math achievement.^77^ Prior studies have found that science and mathematics teachers are more likely to encourage boys to ask and answer questions, explain concepts to boys, praise boys, and spend more time interacting with boys.^22–25^ Another source for gender differences includes parental perceptions of children’s abilities.^26^ Parents who believed that men are superior at math gave significantly higher math-ability estimates to their sons than to their daughters even when controlling for the children’s actual scores.^27,28^ Several studies have also found that parental expectations for children’s abilities and success are correlated with their children’s self-concepts of their own abilities and later performance.^26,29^ In fact, parental perceptions of children’s abilities may influence children’s beliefs in their abilities more than grades.^78^ In addition, teachers’ perceptions of students’ math ability have been shown to predict later math achievement scores when earlier measures of ability are controlled.^21^ Taken together, there is a strong cultural influence on math achievement throughout childhood. Expelling the stereotype that boys have an intrinsic advantage for mathematics in early childhood may lead to increased mathematics exposure and improved parental and societal perceptions, resulting in improved success in mathematics for girls.

The findings presented here provide strong evidence that boys and girls have comparable cognitive faculties for reasoning about mathematics during early childhood. Although it remains possible that gender differences in STEM involvement emerge later in development from maturation,^4^ in other cognitive skills,^79,80^ or from interactions between cultural stereotypes, training, and sexually dimorphic behaviors,^4,81,82^ there is compelling evidence that males’ and females’ abilities are shaped by different cultural experiences that affect their self-image, treatment, and opportunities, and little evidence to support claims of intrinsic or biological gender differences in early mathematical cognition.

## Methods

### Participants

Five hundred and seven children (256 girls, 251 boys, range = 5 months 13 days to 7.92) contributed 868 measures of quantitative reasoning. Informed consent was obtained from children’s parents and assent was obtained from children aged 7 years and older. Children were rewarded with small toys and stickers, and their parents were compensated for travel expenses.

### Sites

Children were tested across three testing sites: the University of Rochester in Rochester, NY, the University of Pittsburgh in Pittsburgh, PA, and Duke University in Durham, NC. Protocols were approved by the local Institutional Review Board at each location and parents of all children provided written consent.

### Testing procedures

#### Preferential looking paradigm for numerosity perception

Eighty children (38 girls, 42 boys, boys’ mean age = 6 months 1 day, girls’ mean age = 6 months and 2 days, range = 5 months 13 days to 6 months 17 days) participated in one of five conditions (16 infants per condition). For full details on additional infants who were tested but excluded from the analyses see previously reported work.^35^

Infants were seated on a parent’s lap or in a high chair approximately 105 cm away from the middle of three 17-inch computer screens. The experimenter began the trials when the infant looked at the attractor on the middle screen (see Fig. 2). Infants completed four 60-s trials. During each trial, infants were simultaneously presented with image streams on each of the two outer computer screens. One stream continuously alternated between two different numbers of dots (numerosities), while the other stream contained images with a constant number of dots. The numerosities on the alternating stream differed by one of five ratios. Infants were randomly assigned to one of these five conditions: 24 vs. 6 (4:1 ratio), 18 vs. 6 (3:1 ratio), 20 vs. 10 (2:1 ratio), 16 vs. 8 (2:1 ratio), and 18 vs. 12 (3:2 ratio). Each image was presented for 500 ms followed by 300 ms of blank screen. Every other image was the same between the two streams, and identical images were interspersed with images that differed in numerosity. One-third of the images that differed between the two streams were matched on either total surface area, individual element size, or total perimeter of the dots. Because half of the images differing in numerosity were matched on density, the two streams could not be differentiated using element size, cumulative surface area, cumulative perimeter, or density. The side of the changing image stream alternated between trials, and the order was counterbalanced between participants. Half of the participants in each condition saw a non-changing image stream containing the larger numerosity, while the other half saw the smaller numerosity.

Looking behavior was recorded digitally and was later coded by an experienced observer using a custom-made coding program written in RealBasic.^83^ A second observer coded more than one-fourth of all participants. Reliability between the two observers was very high (*r* = 0.99). For each stream, we calculated the proportion of time each infant spent looking at the changing and non-changing image streams as a function of total looking behavior to both screens for each infant. The analyses presented here were then conducted on preference scores, which were calculated by subtracting the average percent looking time to the non-changing stream from the percent looking time to the changing stream across all four trials, such that a positive score indicates a preference for changing over non-changing streams.

#### Numerosity discrimination task

Two hundred and fifty children (129 girls, 121 boys, girls’ mean age = 5.43 years, boys’ mean age = 5.46 years, range = 3.07–7.92 years) completed a computerized numerical discrimination task to measure the acuity of their numerosity perception. During the task, children were shown two side-by-side dot arrays too brief to count and were asked to indicate which array contained more dots. Dots varied in location from trial to trial and correct answers (i.e., the larger quantities) were equally presented on the left and right sides of the screen. Children completed one of four versions of this task:

##### Version A

Dot arrays consisted of 1 to 30 dots. Comparisons were defined as having a small, medium, or large number of dots and were made across five different ratios (24 trials per ratio): 4.0 (e.g., 16 dots vs. 4 dots), 3.0, 2.0, 1.43, and 1.11. To ensure that participants used numerical information to make their decision, on half of the trials, the dots were the same size in both arrays, and on the other half of the trials. the dots varied in size between the arrays such that cumulative surface area was the same in both arrays. Dot densities (average interitem distance) varied equally between a large density and a small density.

##### Version B

Dot arrays contained between 12 and 36 dots, and dot size varied within single arrays (average dot diameter = 36 pixels; allowed variation = 20%). There were 18 trials for each of four ratio categories: 2.0, 1.5, 1.17, and 1.14. To ensure that participants used numerical information instead of other perceptual cues to determine the correct response, three trial types were included: Congruent (i.e., the array with the larger number had the larger cumulative area), Incongruent (i.e., the array with the smaller number had the larger cumulative area but both arrays had equal cumulative perimeter), and Neutral (i.e., the arrays had equal cumulative area).

##### Version C

Dot arrays contained 3 to 31 dots and dot size varied within single arrays. There were three ratio categories: 2.0 (easy trial), 1.2533 (medium trial), and 1.11 or less (difficult trial). Children who were successful on the first ten trials (accuracy >80%) completed more medium or difficult trials in the remaining trials to ensure children stayed motivated across the task. Children completed between 30 and 48 trials. In this version, after children made a decision, they placed a bet on how sure they were of their answers. Correct answers were reward with virtual tokens and incorrect answers resulted in taking away tokens.

##### Version D

Dot arrays contained between 3 and 20 dots. Comparisons were made across three ratios (24 trials per ratio): 4.0, 2.0, and 1.25. On ¼ of the trials, dot size was held constant between the two arrays. On another ¼ of the trials, cumulative surface area was constant between the arrays. On ½ the trials, the cumulative surface area varied at a ratio of 2.5 such that on half of those the larger cumulative surface area was congruent with the correct answer and on the other half the larger surface area was incongruent.

To assess the acuity of their numerosity perception, Weber fractions were calculated following Pica et al.^66^ Children were excluded from analyses if their performance on the task was at or below chance (*w* > 74, based on simulated 50% accuracy data; *n* = 1 girl, 2 boys) or if their weber fraction was >2 s.d. from the remaining mean (*w* > 3.99, *n* = 3 girls, 3 boys). The final sample consisted of data from 241 children (125 girls, 116 boys, girls’ mean age = 5.47, boys’ mean age = 5.49). Of these children, data from 32 children who completed version A and 36 children who completed version C had been previously reported, respectively^31,32^ (sample sizes in previous work differ due to differences in age ranges examined in previous vs. present analyses or because some children had also completed the task at an earlier date—we only report the earliest test date in the present work).

#### “How High?” Task

One hundred and forty-three children (71 girls, 72 boys, girls’ mean age = 4.04 years, boys’ mean age = 4.15 years, range = 2.98–5.46 years) completed a rote memorization counting task where they were asked to count out loud as high as they could up to 100. Performance was measured as the highest number counted with no errors.

#### Give-N Task

One hundred and twenty-three children (65 girls, 58 boys, girls’ mean age = 3.83 years, boys’ mean age = 3.92 years, range = 2.98–5.47 years) completed a test of knowledge of number word meanings.^69,70^ In our version of this task, children were asked to remove a specified number of gold coins from a treasure chest, place them on the table, and count them out loud one at a time. Children were allowed to correct their mistakes until they verbally confirmed that the requested number of coins was on the table. The trial was scored as correct or incorrect based on the final number of coins produced. Children were tested on 1 to 10 items, starting with 1 item and continuing until they failed to correctly produced a requested quantity on 2 out of 3 trials of that quantity. If the correct amount was produced, on the next trial, the child was asked to produce a set size of one more than the previous set. If the amount produced was incorrect, then the child was asked for a set size of one less than the previous set. If the child reached 10 items, trials alternated between 9 and 10 until three trials of each set size were administered. Performance was measured as the highest number that was correctly produced on at least two out of three trials.

#### Test of Early Mathematics Ability Third Edition

Two hundred and seventy-five children (142 girls, 133 boys, girls’ mean age 5.47 years, boys’ mean age = 5.43 years, range = 3.07–7.92 years) were administered the TEMA-3,^73^ a comprehensive test of mathematic ability for children aged 3 to 9 years. Questions on the TEMA are classified as formal or informal. Informal concepts draw on untrained, acquired knowledge (e.g., “Show me 3 fingers.”), whereas formal concepts are formally taught in school (e.g., showing an Arabic numeral to the child and asking “What number is this?”). Following standard administration rules, children began at different points on the TEMA depending on their age and continued with the test until five consecutive items were answered incorrectly. Items before the starting point were only administered if the child did not reach five consecutive items that were answered correctly prior to reaching the ceiling point. Overall performance was based on raw scores, which were calculated by first finding the farthest point in the test where five consecutive items were answered correctly. Children then received a point for every question up through that series of five items. This means that children automatically received credit for every question up to the point where correct answers became irregular (<5 consecutive correct items), even for questions that were before the first tested item. An additional point was added to children’s scores for each item answered correctly after that point. Formal and informal scores were the average score (proportion correct) in each category. Unlike the overall performance scores, children were not given credit for formal and informal items that were not administered (e.g., items before the first item tested). Data from 77 children have been previously reported *n* = 10,^33^ *n* = 23,^32^ *n* = 44.^34^

### Analyses

Statistical tests were performed using R (version 3.3.1) and R-Studio (version 1.0.44). Independent-samples *t* tests were conducted using the “ttest” function assuming equal variance. Tests of equivalence were conducted using the “TOSTtwo.raw” function from the TOSTER package (upper and lower bounds set to ±0.5 × standard deviation of the entire group; *α* = 0.05). This function returns two *t* values (*t*_1_ and *t*_2_). For statistical equivalence, both *t* values must be statistically significant. Statistical equivalence is rejected if either *t*_1_ or *t*_2_ does not reach significance. Regressions were conducted using the “lm” function. Levene’s test of equivalence was carried out using the “leveneTest” function in the car package. ANOVAs were conducted using the “ezANOVA” function (type = 3) from the ez package.

Growth curves were calculated at the group level.^40^ Specifically, Eq. 1 was fit to the data using the nls function (“port” algorithm) in R. Equation 1 fits two free parameters, alpha (*α*), which indicates the rate of growth, and lambda (*λ*), which indicates the age at which children are halfway to the maximum possible score. In Eq. 1 *β*_0_ and *β*_1_ represent the lower and upper score limits and are set to the lowest and highest possible scores for a given measure before the free parameters are fit to the data.

1 $${\mathrm{Quantitative}}\,{\mathrm{Reasoning}}\,{\mathrm{Ability}} = \beta _0 + \frac{{\beta _1 - \beta _0}}{{1 + {\mathrm{e}}^{ - \alpha \left( {{\mathrm{Age}} - \lambda } \right)}}}.$$

The growth curves displayed in Figs. 3–5 were calculated across the group data, and the standard deviation (shaded areas) at each age was calculated across 10,000 growth curves fit by bootstrapping the data. Because these curves were fit at the group level rather than the individual level, no statistical tests of gender-specific growth rates were conducted. Instead, these growth curves are meant to simply provide a visualization of boys’ and girls’ performance within the age range of the children in our sample.

### Data availability

The datasets analyzed during the current study are available from the corresponding author on reasonable request.

## Electronic supplementary material

Supplementary Notes

## Electronic supplementary material

**Supplementary Information** accompanies the paper on the *npj Science of Learning* website (10.1038/s41539-018-0028-7).
