# Supplementary material for: No intrinsic gender differences in children’s earliest numerical abilities
Source: NPJ Sci Learn. 2018 Jul 6;3:12. doi: 10.1038/s41539-018-0028-7 (PMC6220191; doi:10.1038/s41539-018-0028-7)
Supplement: Supplementary file 1 — Supplementary Notes [file 41539_2018_28_MOESM1_ESM.pdf]

Supplement 1: Analyses controlling for age

Numerosity Perception. Controlling for age in the analyses of 3- to 7-year-olds' Weber fractions revealed the same patterns of results: Schuirmann's Tests of Equivalence revealed statistical equivalence ($t_1(239) = 4.05$, $p = 0.00003$; $t_2(239) = -3.69$, $p = 0.0001$) and Levene's Test of Equality of Variance revealed no difference in variance between boys and girls ($F(1,239) = 0.10$, $p = 0.75$, boys' sd = 0.50, girls' sd = 0.39).

Recitation of Count List. Controlling for age revealed the same pattern of results on the 'How High?' task as reported before. Schuirmann's Tests of Equivalence revealed statistical equivalence ($t_1(141) = 4.14$, $p = 0.00003$; $t_2(141) = -1.84$, $p = 0.03$). Levene's Test of Equality of Variance did not reveal a difference in variability between boys and girls ($F(1,141) = 1.81$, $p = 0.18$; boys' sd = 26, girls' sd = 20).

Knowledge of Number Word Meanings and Counting Principles: When controlling for age Schuirmann's Tests of Equivalence still did not reveal statistical equivalence ($t_1(121) = 4.33$, $p = 0.00002$; $t_2(121) = -1.24$, $p = 0.11$). Levene's Test of Equality of Variance did not reveal a significant difference in variance ($F(1,121) = 0.65$, $p = 0.42$; boys' sd = 2.87, girls' sd = 2.72).

Early Childhood Math Achievement. When TEMA scores were controlled for age, Schuirmann's Tests of Equivalence continued to reveal statistical equivalence, and there was no difference in variability (equivalence tests: $t_1(273) = 6.71$, $p < 0.00001$; $t_2(212) = -1.66$, $p = 0.048$; test of equality of variance: $F(1,273) = 2.37$, $p = 0.13$; boys' sd = 9.61, girls' sd = 8.65). A 2 x 2 (gender x question type) ANOVA performed on age-controlled scores also did not reveal an interaction between gender and question type on proportion correct controlled for age (formal vs informal; Gender: $F(1,207) = 2.27$, $p = 0.13$; Question Type: $F(1,207) = 0.0004$, $p = 0.98$; Gender x Question Type: $F(1,207) = 0.39$, $p = 0.53$) and boys and girls continued to show statistical equivalence and no difference in variance on both formal and informal questions (Formal Questions: equivalence tests: $t_1(207) = 4.12$, $p = 0.00003$; $t_2(207) = -3.09$, $p = 0.001$, boys' mean = 0.006, girls' mean = 0.006; variance test: $F(1,207) = 0.0004$, $p = .63$, boys' sd = 0.17, girls' sd = 0.17; Informal Questions: equivalence tests: $t_1(207) = 5.23$, $p < 0.00001$; $t_2(207) = -2.03$, $p = 0.02$, boys' mean = 0.01, girls' mean = 0.02; variance test: $F(1, 207) = 0.01$, $p = 0.93$; boys' sd = 0.14, girls' sd = 0.14).

Supplement to: Kersey, Braham, Csumitta, Libertus, & Cantlon. No intrinsic gender differences in children's earliest numerical abilities. *npj Science of Learning.*
